# TGF-Net: Transformer and gist CNN fusion network for multi-modal remote sensing image classification

**DOI:** 10.1371/journal.pone.0316900

**Published:** 2025-02-19

**Authors:** Huiqing Wang, Huajun Wang, Linfen Wu

**Affiliations:** 1 Center for Information and Educational Technology, Southwest Medical University, Luzhou, Sichuan, China; 2 School of Geophysics, Chengdu University of Technology, Chengdu, Sichuan, China; 3 College of Health and Intelligent Engineering, Chengdu Medical College, Chengdu, Sichuan, China; Xidian University, CHINA

## Abstract

In the field of earth sciences and remote exploration, the classification and identification of surface materials on earth have been a significant research area that poses considerable challenges in recent times. Although deep learning technology has achieved certain results in remote sensing image classification, it still has certain challenges for multi-modality remote sensing data classification. In this paper, we propose a fusion network based on transformer and gist convolutional neural network (CNN), namely TGF-Net. To minimize the duplication of information in multimodal data, the TGF-Net network incorporates a feature reconstruction module (FRM) that employs matrix factorization and self-attention mechanism for decomposing and evaluating the similarity of multimodal features. This enables the extraction of distinct as well as common features. Meanwhile, the transformer-based spectral feature extraction module (TSFEM) was designed by combining the different characteristics of remote sensing images and considering the problem of orderliness of the sequence between hyperspectral image (HSI) channels. In order to address the issue of representing the relative positions of spatial targets in synthetic aperture radar (SAR) images, we proposed a spatial feature extraction module called gist-based spatial feature extraction module (GSFEM). To assess the efficacy and superiority of the proposed TGF-Net, we performed experiments on two datasets comprising HSI and SAR data.

## 1. Introduction

Relevant ground features are effectively captured in remote sensing(RS) through the utilization of hyperspectral images (HSI), multispectral images (MSI), light detection and ranging (LiDAR), as well as SAR images, which enable the extraction of unique characteristics. HSI have rich spectral characteristics and can distinguish ground objects with similar textures and different spectra. The MSI effectively capture and represent the color, brightness, and distinctive features of various ground objects, thereby exhibiting remarkable recognition capabilities in urban environments encompassing streets, buildings, water bodies, soil compositions, and vegetation types. The SAR images primarily capture two types of ground target characteristics: structural attributes (such as texture and geometry) and electromagnetic scattering properties (including dielectric and polarization features). In theory, the utilization of complementary information from multi-modal RS images has the potential to enhance feature classification accuracy and compensate for limitations associated with single-modal images. By merging data from various modalities, a more comprehensive feature representation can be constructed, enabling the acquisition of detailed information [1]. This method of analyzing remotely captured images offers numerous advantages in various research and practical fields, such as categorizing land usage [2], identifying targets, segmenting objects based on their meaning [3], monitoring the environment, exploring mineral resources [4], planning urban areas, diagnosing medical conditions [5], implementing precise agricultural techniques, and ensuring food security [6]. It is worth noting that land usage categorization has gained significant attention as a particularly prominent application area. Therefore, the utilization of multi-modal RS image classification holds significant research value. Extensive research has been carried out to develop successful HSI and SAR fusion models, which fall into two main categories. The first category includes traditional classification algorithms such as morphological (MP) and subspace learning ones. Ghamisi et al. [7] suggested an alternative method for land cover classification by jointly extracting attribute profiles (APs), rather than MP, obtained from the combination of HSI and LiDAR data sources. Xia et al. [8] evolved an integrated classifier for classification tasks using HSI and LiDAR. However, this method has a high computational overhead despite the improvement in classification accuracy. Hu et al. [9] conducted an in-depth study on topological analysis theory and proposed a semi-supervised fusion classification algorithm based on mapper framework. Through comprehensive analysis and visualization of complex structures in high-dimensional data sets, multi-modal data were efficiently integrated to achieve accurate classification of land use and cover. Hong et al. [10] proposed a subspace-based method for spectral data registration and classification. The graph is constructed by the similarity between samples and the subspaces of different categories, and then the subspace is aligned to improve the classification accuracy of the data. However, the utilization of shallow feature classification models in these approaches poses challenges in effectively handling intricate sample data and nonlinearities. Furthermore, enhancing classification accuracy is hindered by the heavy reliance on a priori information within these methods.

The second model is a deep learning-based fusion approach. In an integrated approach, deep learning networks acquire valuable characteristics progressively, starting from lower levels and advancing towards higher ones. Researchers have proposed various multimodal RS data classification methods. These methods have been utilized for RS image classification, surpassing the performance bottleneck of using single channels and achieving better results. In their study, The accuracy of classification can be enhanced by utilizing a CNN that combines feature-level fusion and decision-level fusion techniques, as suggested by Hang et al. [11]. Hong et al. [12] put forward an encoder-decoder network structure named EndNet, which proves to be a straightforward and efficient approach for classifying HSI and LiDAR data. As per Gadiraju et al. [13], an effective method was proposed to classify crops with exceptional accuracy by integrating multispectral and multitemporal satellite images using a multimodal deep learning approach. The research conducted by Suel et al. [14] utilized diverse deep learning methods to assess urban traffic congestion, estimate the economic status of cities, and forecast potential ecological harm through the integration of RS data and street view imagery. Zhang et al. [15] introduced a novel cross-aware CNN model for integrating diverse data types and enhancing the accuracy of joint classification. Despite the considerable advancements achieved by these deep learning techniques in CNNs, they have greatly enhanced the classification performance of HSI and LiDAR data, however, the limited training data and feature redundancy lead to a relatively high computational cost. Hong et al. [16] proposed a new classification method based on spectral feature extraction of transfer encoder (TE) module across different layers, which can integrate spectral features of different layers and can effectively capture adjacent band information. However, their work focuses solely on processing spectral data using transmission techniques. Although spectral features can be successfully captured, spatial information cannot be exploited. Roy and colleagues [17] proposed a fusion transformer that integrates HSI and LiDAR data to achieve joint classification. The approach involves utilizing LiDAR data as trainable tokens to facilitate feature learning alongside HSI tokens. Liu et al. [18] proposed a new deep group spatial-spectral attention fusion network (GAFNet). The network effectively extracts and combines low-level and high-level features. The global abstract information and local detail information are preserved in the HSI classification process, and the classification accuracy is improved. A novel network with adaptive hybrid fusion was proposed by Ma et al. [19] for classifying remote sensing images at multiple resolutions. The network combined CNN and multi-resolution transform (MRT) technology, and used an adaptive fusion strategy to fuse the features of CNN and MRT. A multi-scale progressive collaborative attention network for classification of fused RS images was proposed by Ma et al. [20]. The network adopts a novel progressive attention mechanism, as well as a multi-scale feature extraction and collaboration mechanism, which can effectively fuse multi-source RS images and improve the classification accuracy. In their study, Li et al. [21] proposed a graph-based self-supervised learning framework for hyperspectral and multispectral data fusion classification network, namely GFESAN classification network. The GFESAN network improves the discrimination ability of the network by assigning the most appropriate class label to each pixel, and the multi-scale feature aggregation strategy obtains the local and global level environmental information. The LMF-KJSR method, proposed by Chen et al. [22], introduces a novel approach for HSI classification. By leveraging the joint sparse representation framework and incorporating spatial information extracted from local matrix features, this method enhances both the accuracy and robustness of HSI classification. Additionally, it effectively combines spectral and spatial information to achieve superior results in classification tasks. Zhang et al. [23] proposed a tree species classification method based on morphological transformation and spatial logic aggregation, namely MTSLA classification method. This method enhances the spectral differences between different tree species by morphological transformation, and integrates spatial information and logical constraints into the classification process by spatial logic aggregation, so as to realize the fusion of spatial data and spectral data, so as to improve the accuracy of tree species classification. Zou et al. [24] proposed a multi-scale residual classification network based on dual attention enhancement, namely DA-IMRN network. The network enhances the information interaction between these two branches by using the bidirectional attention mechanism, and uses the multi-scale spectral and spatial residual blocks to extract the feature information of different receptive fields, so as to improve the quality of the feature map, Finally, the spectral-spatial information was fused to improve the classification accuracy. Wang et al. [25] proposed a new multi-level self-guided classification network based on image asymmetric information and feature attention mechanism. The network enhances the discrimination between object similarity and background difference samples through object-background separation, and identifies common features in samples in a self-guided manner to ensure the consistency of feature representation. At the same time, texture and morphological features are combined to guide feature learning, thereby reducing the differences between categories and achieving improved classification accuracy. Wang et al. [26] proposed a multimodal RS image classification method based on CNN cross-modal fusion and reconstruction, namely CMR-Net. The network structure still takes CNN as the base network, and a cross-modal fusion and reconstruction module is designed in multimodal feature fusion, which can cross-fuse the feature information of the two modalities to effectively extract the spectral-spatial feature information of multimodal data. The effectiveness of this classification method is demonstrated through experiments on the Houston 2013 data set and the Berlin data set to improve the classification accuracy of multimodal remote sensing data. Experiments on the Houston 2013 dataset and the berlin dataset demonstrate the effectiveness of the classification method and improve the classification accuracy of multimodal remote sensing data. However, the existing multimodal remote sensing image classification methods mentioned above insufficiently consider the redundant information across diverse modalities, the sequential ordering of HSI channels, and the relative positional representation of spatial targets in SAR images. These factors can significantly impact both the accuracy of classification and interpretation of multimodal remote sensing images.

To tackle the previously mentioned concerns, this study introduces a fusion network that combines transformer and gist CNN for classifying multi-modal remote sensing images. It is called TGF-Net for short. The effectiveness of our method is corroborated by the experimental outcomes presented in the present investigation. The primary achievements of this study can be summarized in the following manner.

1) Aiming at the problem of information redundancy between multi-modal data, a feature reconstruction module (FRM) based on matrix factorization and self-attention mechanism was proposed. This module calculates the matrices of similarity and contrasting similarity by reconstructing features is achieved by factorizing the matrix of coefficients. In this way, redundant information between common features can be eliminated and unique features can be characterized.

2) A transformer-based spectral feature extraction module (TSFEM) is proposed to address the problem of ordering the sequences of HSI channels. This TSFEM module removes the noisy channels through a feature selection mechanism and subsequently employs the transformer encoder to derive distinctive characteristics from the channel sequence. This module simplifies the process of obtaining features in a specific sequence.

3) To address the issue of relative position representation of spatial targets in SAR images, this paper introduces the gist-based spatial feature extraction module (GSFEM). This module introduces gist features to extract various spatial location features of SAR images by means of CNN, and at the same time characterizes the relative position information of spatial features.

## 2. Methods

This section provides a comprehensive explanation of the proposed approach. Initially, we present an overview of the fusion network’s structure, which combines transformer and gist CNN techniques. Then, a feature reconstruction module (FRM) based on matrix factorization and self-attention mechanism was proposed. Secondly, In the phase of extracting features, according to the characteristics of different RS images, different feature extraction modules are designed, which a transformer-based spectral feature extraction module, namely TSFEM, and the gist-based spatial feature extraction module, namely GSFEM. The specifics are outlined below.

### 2.1 Architecture for network fusion of transformer and gist CNN features

**Fig 1:**
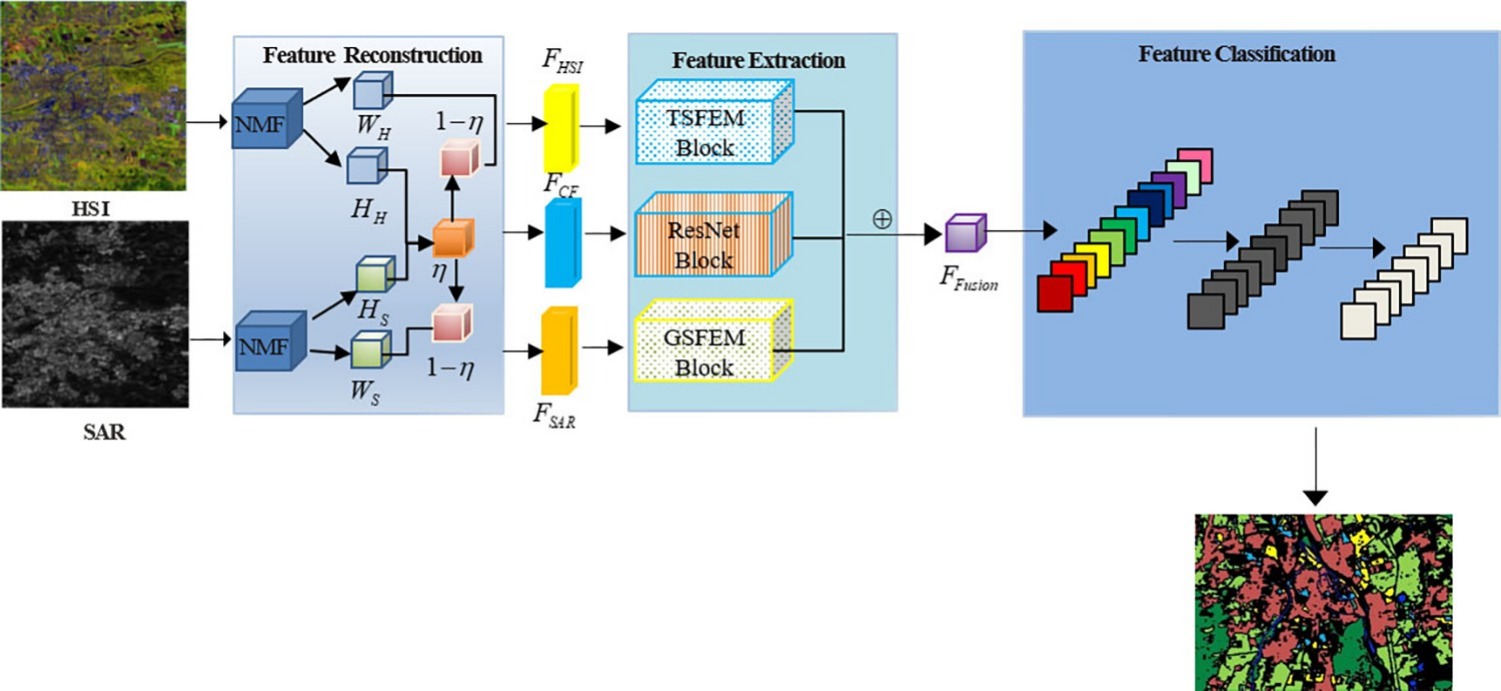
Network architecture based on transformer and gist CNN feature fusion (It consists of feature reconstruction, feature extraction and feature classification, where *η* is correlation matrix, the TSFEM is used for unique spectral feature extraction, the GSFEM module is used for unique spatial feature extraction, and the ResNet module extracts common features).

The structure of the network proposed in this study, as shown in Fig 1, consists of three main parts: feature reconstruction, feature extraction, and feature classification. At the beginning, to decompose features, we utilize the FR which combines matrix factorization and self-attention mechanism, as depicted in Fig 1. Subsequently, we designed diverse feature extraction modules for various branches. A solitary spectral feature is extracted utilizing the TSFEM, as demonstrated in Fig 3. The GSFEM is employed for the extraction of individual spatial features, as illustrated in Fig 4. Common features are extracted by means of the resnet block [27]. In the end, feature classification is carried out using three layers that the final layer’s count corresponds to The quantity of classification categories. The employed loss function is cross-entropy.

$$loss=-\frac{1}{n_{p}}\sum_{j=1}^{n_{p}}\left[y_{j}\;\log({\hat{y}}_{j})+(1-y_{j})\log(1-{\hat{y}}_{j})\right]$$ (1)

where *n*_*p*_ represents the batch block size, *y*_*j*_ refers to the label of the *j*th input pair, and ${\hat{y}}_{j}$ denotes the category probability of the *j*th input pair. The cross-entropy loss function loss signifies the discrepancy between the actual probability distribution and the forecasted probability distribution. A smaller cross-entropy value signifies a more effective prediction by the model.

### 2.2 Feature Reconstruction (FR)

HSI are rich in spectral data, but SAR images have better spatial detail. They capture different remote sensing modalities in the same region, leading to a substantial amount of redundant information. Therefore, we introduce a FR to eliminate unnecessary data and boost feature representation capabilities. The feature reconstruction module mainly decomposed the HSI and SAR data to obtain the main feature matrix and correlation coefficient matrix, and then carried out feature enhancement calculation, calculated the similar matrix, and obtained the reconstituted feature. The feature reconstruction is depicted in Fig 1.

*1) Principles of the self-attention mechanism*. The conventional methods of incorporating spatial and channel attention can be utilized to capture the features at both local and global levels [28, 29]. Parameter estimation in their approach involves employing gradient descent and backpropagation techniques. Conversely, self-attention model incorporates global statistical information by performing weight updates within the input sequence using its own computations [30]. This approach is more advantageous for describing features on a global scale. The design in question is visually depicted in Fig 4.

First, three different vectors are learned by linearly transforming the feature ***X***∈***R***^*H*×*W*×*C*^ after positional embedding: ***Q***∈***R***^*H*×*W*×*C*^, ***K***∈***R***^*H*×*W*×*C*^ and ***V***∈***R***^*H*×*W*×*C*^. These three vectors are computed by multiplying ***X*** with the ***W***^***Q***^,***W***^***K***^,***W***^***V***^ counterparts respectively. where ***W***^*Q*^,***W***^*K*^,***W***^*V*^∈***R***^*H*×*W*×*C*^. Then ***Q***,***K***,***V*** are randomly initialized as:

$$Q=W^{Q}X,K=W^{K}X,V=W^{V}X$$ (2)

Second, to assess the impact of individual markers on the overall sequence, it is essential to conduct a similarity calculation. We calculate the attention score by taking the dot product of ***K*** and ***Q***. Subsequently, The outcome of the dot product is divided by the initial dimension of ***K***.

This process mitigates the gradient explosion issue. Finally, the output values are compressed into the range of [0,1] using the SoftMax activation function. i.e.:

$$E=softmax(\frac{QK^{T}}{\sqrt{d_{k}}})$$ (3)

**Fig 2:**
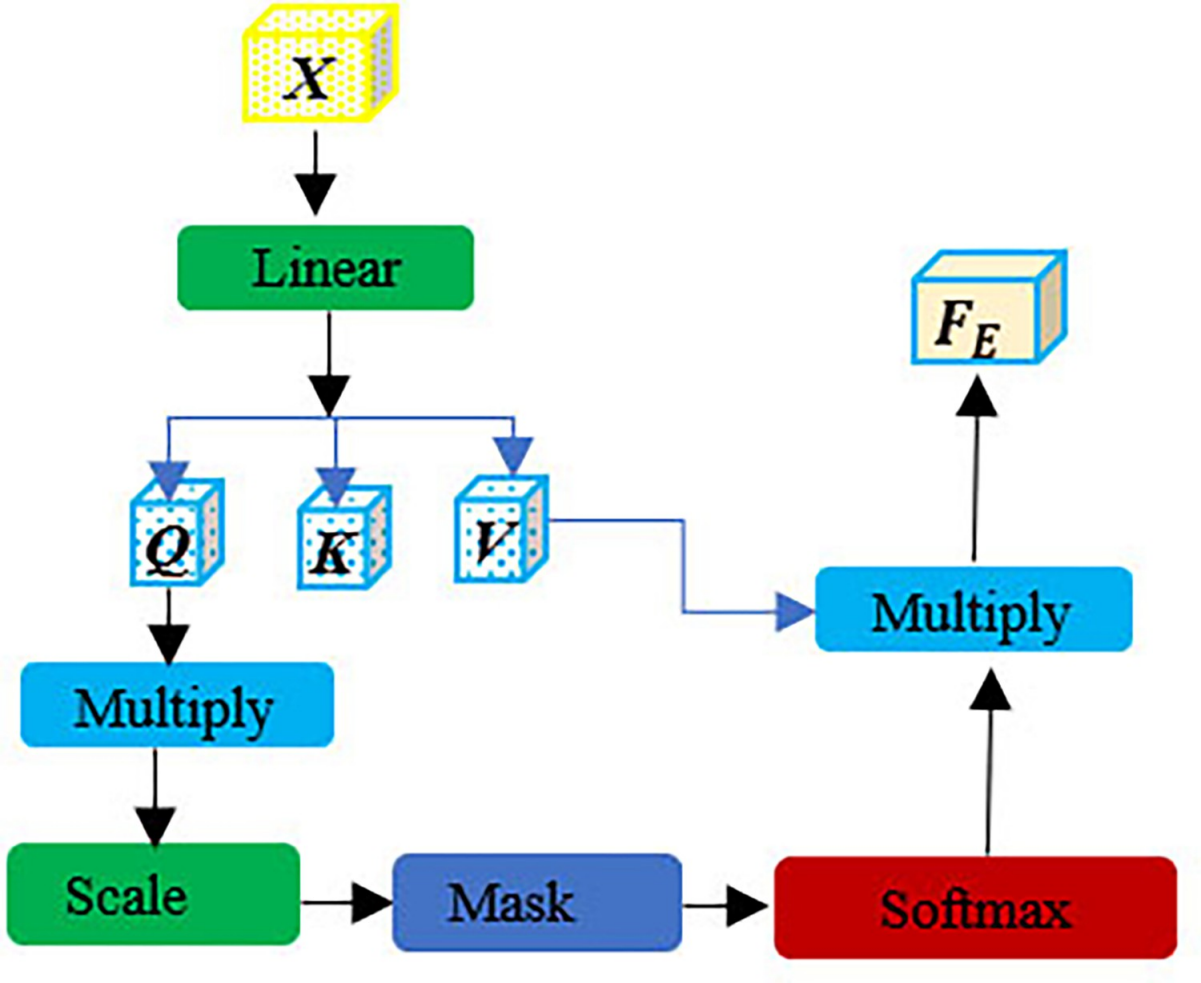
Self-attention module.

Finally, the attributes are amplified, The enhanced feature ***F***_***E***_∈***R***^*H*×*W*×*C*^ is obtained by multiplying ***E*** and ***V***, where ***E***∈***R***^*H*×*W*×*C*^, Among them, Fig 2 shows the calculation process of the self-attention module. The results of the calculations are

$$F_{E}=EV$$ (4)

In summary, the overall principles of the self-attention mechanism is:

$$Attention(Q,K,V)=softmax(\frac{QK^{T}}{\sqrt{d_{k}}})V$$ (5)

Initially applied in natural language processing, self-attention mechanisms have lately found their way into computer vision. Nonetheless, processing feature inputs in chunks could potentially bury useful target information under irrelevant signals, hindering the accurate depiction of features.

*2) Feature decomposition*. Initially, the HSI and SAR image undergo NMF. In this context, **F**_**HSI**_∈**R**^*H*×*W*×*C*^ represents the hyperspectral image, **F**_**SAR**_∈**R**^*H*×*W*×*C*^ denotes the synthetic aperture radar image, where *H* denotes the height of the image, *W* denotes the width of the image, and *C* denotes the number of channels of the image. Both the initial matrix and the two matrices resulting from matrix factorization are positive matrices, and there is only a single factorization method that fulfills the requirements of both existence and uniqueness. This approach can be employed for capturing image attributes, assisting in swift target recognition, and is closely aligned with the way humans perceive visual information. The expressions for **F**_**HSI**_ and **F**_**SAR**_ are as follows:

$$F_{HSI}=W_{h}H_{h}$$ (6)

$$F_{SAR}=W_{s}H_{s}$$ (7)

where **H**_**h**_ and **H**_**s**_ denote the coefficient matrices corresponding to two distinct feature spaces, while **W**_**h**_∈**R**^*H*×*W*×*C*^ stand for the primary characteristic arrays of HSI, **W**_**s**_∈**R**^*H*×*W*×*C*^ represent the primary characteristic arrays of SAR. Each column vector within **W**_**h**_ and **W**_**s**_ corresponds to a principal component of the original features **F**_**HSI**_ and **F**_**SAR**_, and Each column vector in **H**_**h**_ and **H**_**s**_ represents the mapping of the feature space onto the original feature space.

*3) Objective function and similarity computation*. The objective function *f*_obj_ is devised to progressively refine the main feature matrices (**W**_**h**_ and **W**_**s**_) and coefficient matrices (**H**_**h**_∈**R**^*H*×*W*×*C*^, **H**_**s**_∈**R**^*H*×*W*×*C*^) through iterative reconstructions, in order to better align with the original features. The objective function is expressed as:

$$f_{obj}=\min\frac{1}{2}\|F_{HSI}-W_{h}H_{h}{\|}_{2}^{2}+\frac{1}{2}\|F_{SAR}-W_{s}H_{s}{\|}_{2}^{2}+\|W_{h}{\|}_{1}^{2}+\|W_{s}{\|}_{1}^{2}$$

$$W_{h},H_{h},H_{s},W_{s}>0$$ (8)

$$\eta =\frac{{\left(H_{h}-{\bar{H}}_{h}\right)}^{T}(H_{s}-{\bar{H}}_{s})}{\|H_{h}-{\bar{H}}_{h}{\|}_{F}\cdot \|H_{s}-{\bar{H}}_{s}{\|}_{F}}$$

where $\|\cdot {\|}_{2}^{2}$ denotes the square of the L2 norm and $\|\cdot {\|}_{1}^{2}$ denotes the square of the L1 norm. **η**∈**R**^*H*×*W*×*C*^ denotes the relationship between the matrices **H**_**h**_ and **H**_**s**_ in terms of their coefficients, At the same time, ${\bar{H}}_{s}\in R^{H\times W\times C}$ denotes the average values of **H**_**h**_ and **H**_**s**_. ||∙||_*F*_ refers to the Frobenius regularization function.

*4) Self-attentive feature enhancement*. Initially, the self-attention mask scores are computed globally using *softmax*(∙) function, followed by the extraction of enhancement features. Relevant characteristics are obtained by analyzing the correlation matrix *η*, whereas individual channel features are reconstructed based on the anticorrelation matrix 1−*η*. The specific computation procedure is outlined as follows.

$$\begin{array}{l} F_{CF}=softmax(\frac{\eta }{\sqrt{d_{h}}})H_{h}W_{h},\\ F_{HSI}^{N}=softmax(\frac{1-\eta }{\sqrt{d_{\eta }}})H_{h}W_{h},\\ F_{SAR}^{N}=softmax(\frac{1-\eta }{\sqrt{d_{\eta }}})H_{s}W_{s} \end{array}$$ (9)

where *d*_*η*_∈**R**^*H*×*W*×1^, **F**_**CF**_∈**R**^*H*×*W*×*C*^ represents the shared features, $F_{HSI}^{N}\in R^{H\times W\times C}$ refers to the individual spectral feature of HSI, and $F_{SAR}^{N}\in R^{H\times W\times C}$ denotes the individual spectral feature of SAR. The value of N is set to 10. This approach effectively captures the distinctive aspects of both collective and individual characteristics, while simultaneously minimizing the divergence in semantic representation. Consequently, it mitigates the superfluous information across diverse modalities.

The traditional approach to fusion of feature reconstruction involves the partitioning of the original multimodal image into two separate matrices, one for spatial coefficients and another for principal feature matrices. The fused image is generated by combining the principal feature matrix obtained from the HSI with the correlation coefficients derived from the SAR image. In this research, we incorporate the self-attention mechanism to reconstruct the characteristics. The extraction of shared features is achieved by measuring the correlation of the coefficient matrix. This method allows for the separation and reconstruction of shared and unique characteristics using a comprehensive statistical approach, providing greater benefits for subsequent extraction and recognition of features.

### 2.3 Feature extraction module

#### 2.3.1 A transformer-based Spectral Feature Extraction Module (TSFEM)

**Fig 3:**
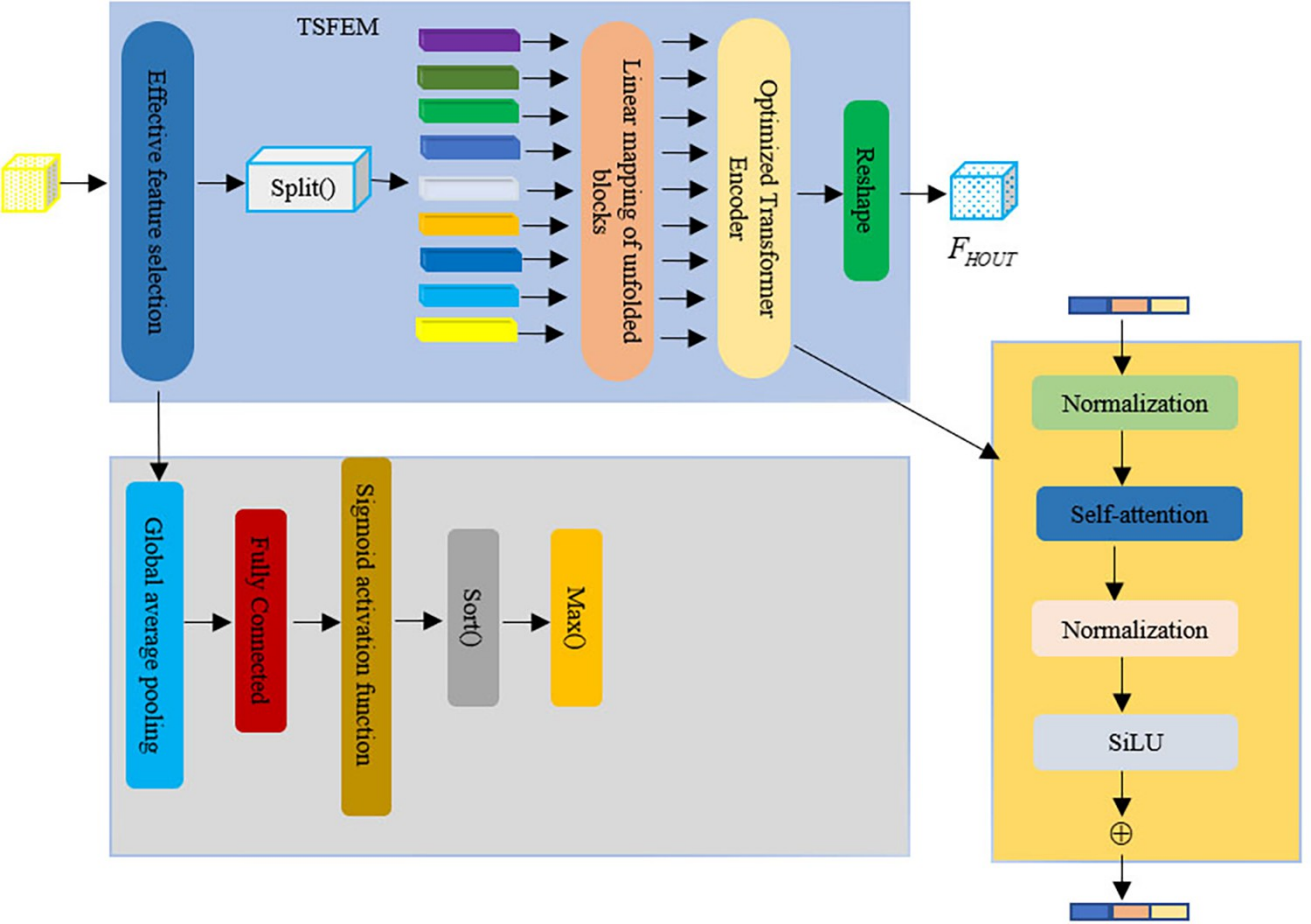
TSFEM spectral feature extraction module (It mainly includes effective feature selection, extended linear conversion module, and optimized transformer coding).

HSI are characterized by their wide spectral bands and high spectral as the particular arrangement of elements connections between spectra. However, It is the high correlation between neighboring bands that increases the duplication of information among them. As a result, an effective feature selection method is employed to identify the essential components of a transformer for extracting global statistical features. The detailed process of feature extraction can be seen in Fig 3.

*1) Effective feature selection*. Hyperspectral features convey significant channel information. Nonetheless, a bulk of these channels are grouped on insignificant backgrounds, which could potentially cause the target features of interest to get lost in distracting signals. Consequently, we opt for the primary channel of interest as the transformer input.

First, the input features undergo a pooling operation that utilizes global averaging to compact the spatial information. Subsequently, vector features are extracted utilizing the full connectivity layer (FC) to enhance the interaction amongst the channel streams. In the second stage, the self-attention is derived through the sigmoid activation function for subsequent feature selection and amplification. Its output weight vector is calculated as follows:

$$\omega =\delta (f_{FC}(g(F_{in})))$$ (10)

In this study, we utilize ***F***_***in***_∈**R**^*H*×*W*×*C*^ to represent input characteristics. The pooling is denoted by *g*(∙), while *f*_*FC*_(∙) represents a fully connected layer. Additionally, *δ*(∙) signifies a sigmoid activation function and ***ω***∈**R**^1×1×*C*^ refers to an output weight vector.

Second, to enhances channel selection efficacy and resilience, we employ *sort*(∙) for arranging attention weights in descending order along with recording their original indices prior to sorting. Furthermore, max(∙) aids in selecting top-k channels as prominent features. The calculation process remains unchanged.

$$\omega^{'},p=\max(sort(\omega ))$$ (11)

where *sort*(∙) is the sorting function, ***ω***′∈**R**^1×1×*K*^ is the vector of max(∙), and ***p*** is the index of the top-k channels.

Finally, the original feature ***F***_***in***_ contains the corresponding channel feature $F_{in}^{'}$ based on index ***p***. Additionally, we perform a multiplication operation involving the feature $F_{in}^{'}$ and the chosen weight vector ***ω***′. formula can be expressed as follows:

$$F_{h}=\omega^{'}F_{in}^{'}$$ (12)

where ***F***_***h***_∈**R**^*H*×*W*×*K*^, which diminishes the dimensionality of the input characteristics and additionally enhances the excellence of the input features.

*2) Extend the linear transformation of the blocks*. The divisions of each channel in the prominent features are employed as inputs for the transformer encoding. These inputs are subsequently combined in the spatial dimension and converted into vectors through a fully connected layer. To be specific:

$$\upsilon =f_{FC}(split(F_{h}))$$ (13)

where ***υ***∈**R**^*H*×*w*×*K*^, *split*(∙) is the channel splitting function, which guarantees the identification of subsequent overall statistical attributes.

*3) Optimize transformer encoding*. Input of characteristics into the transformer encoding module. In this research, we develop a surface-level network and introduce the SiLU function as an alternative to the activation function, which is also known as the Sigmoid-Weighted-Linear-Unit (SiLU). This function demonstrates nonlinearity and absence of upper and lower bounds, exhibiting both smoothness and non-monotonicity simultaneously. The detailed calculations are as follows:

$$F_{HS}=LN(\upsilon +SA(LN(\upsilon )))+f_{FC}(SiLU(f_{FC}(LN(\upsilon +SA(LN(\upsilon )))))$$ (14)

where *LN*(∙) denotes the normalization layer and *SA*(∙) denotes the self-attention. Finally, ***F***_***HS***_∈**R**^*H*×*w*×*K*^ serves as a matrix for the SSFE-Module output. Namely:

$$F_{HOUT}=reshape(F_{HS})$$ (15)

where **F**_**HOUT**_∈**R**^*H*×*w*×*K*^, *reshape*(∙) denotes the shape transformation function.

The TSFEM considers the serial connection between HSI channels and proposes a notable channel selection approach to minimize redundant data in the transformer input [29]. This not only highlights the advantageous multi-channel characteristic of HSI images, but also eliminates redundant channels and improves feature depiction.

#### 2.3.2 Gist-based spatial feature extraction module (GSFEM)

**Fig 4:**
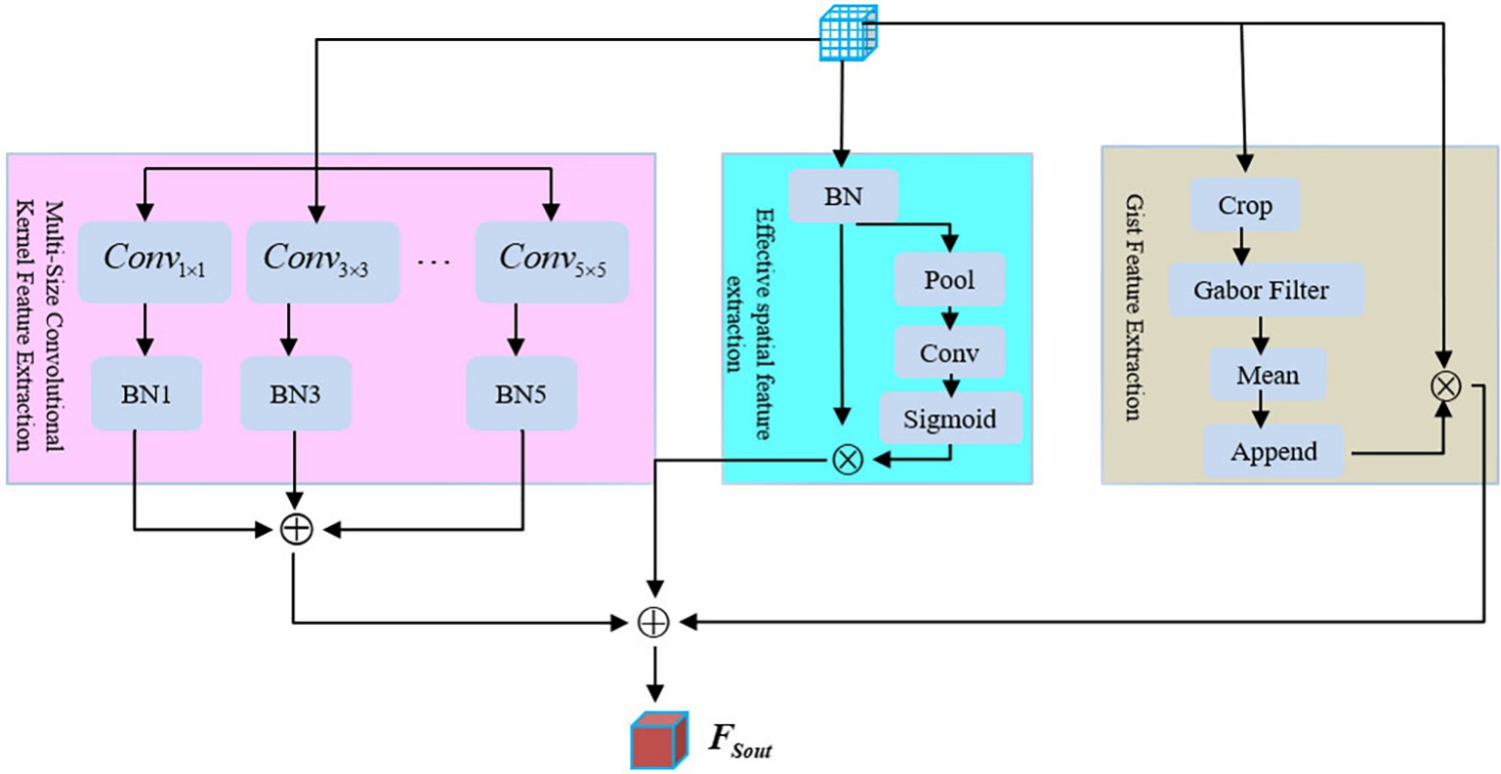
Gist-based spatial feature extraction module (It mainly includes multi-scale feature extraction, effective spatial feature extraction and gist feature extraction).

In comparison to HSI, SAR images possess more comprehensive spatial data and a greater disparity in target sizes. Consequently, gist-based spatial feature extraction module, referred to as GSFEM, is devised in this study, as illustrated in Fig 4.

*1) Multi-scale feature extraction*. Feature extraction through multi-scale convolutional kernels in RS images at a large scale. This module addresses the significant variations in target size observed in such images. It employs the use of three distinct convolution kernels, each operating at a different scale, for the extraction of diverse characteristics. These characteristics are subsequently merged through the process of summation.

$$F_{smcout}=BN_{1}(Conv_{1\times 1}(F_{in}))+\cdots BN_{3}(Conv_{3\times 3}(F_{in}))\cdots +BN_{5}(Conv_{5\times 5}(F_{in}))$$ (16)

where ***F***_***in***_∈**R**^*H*×*w*×*K*^ is the input feature, *Conv*_*n*x*n*_(∙) denotes a convolutional kernel of size *n*, *BN*_*n*_(∙) denotes batch regularization, and ***F***_***smcout***_∈**R**^*H*×*w*×*C*^ denotes a high-resolution output feature.

*2) Effective spatial feature extraction*. The semantic representation capabilities of images vary based on their spatial locations. Consequently, we extract the weights ***ξ***∈**R**^*H*×*w*×*C*^ for each space within the input feature map. Subsequently, these weights are multiplied with the normalized input features, as follows:

$$\begin{array}{l} \xi =\delta (Conv_{1\times 1}(op_{sq}(BN(F_{in}))))\\ F_{seout}=\xi BN(F_{in}) \end{array}$$ (17)

where *op*_*sq*_(∙) represents the process of channel compression. ***F***_***seout***_∈**R**^*H*×*w*×*C*^ stands for the active spatial output features, which establish links among different areas, thus increasing the dimensionality of the relevant feature space for the task and reducing the impact of feature spaces with negligible influence on the classification assignment.

*3) Extracting gist feature*. Typically, CNNs struggle with identifying the spatial connections among objects and face limitations when it comes to extracting specific details about their relative positions. Nevertheless, for scene classification purposes, it becomes crucial to establish robust associations between these localized characteristics; hence the introduction of gist features. These particular attributes emulate human vision by capturing contextual information within an image using a multi-scale multi-directional filtering technique referred to as the Gabor filter array *gf*_*a*_(∙). First, the input characteristics can be categorized into a grid of 4×4 areas. Next, we apply Gabor filters to segment the characteristics. Subsequently, we compute the average of the filtered attributes within each spatial window for every region and combine them into vectors of size 1×1×*C* for each window. Finally, we perform a multiplication operation between these vectors and the initial input features in order to derive the resulting gist features, namely:

$$F_{Gistout}=(f_{append}(f_{mean}(gf_{a}(f_{crop}(F_{in})))))\cdot F_{in}$$ (18)

where *f*_*crop*_(∙) is the meshing operation, *f*_*mean*_(∙) denotes the function to find the mean, *f*_*append*_(∙) denotes the cascade operation, ***F***_***Gistout***_∈**R**^*H*×*w*×*C*^ denotes the gist feature output.

*4) Feature fusion*. Relevant spatial enhancement and gist features are combined using summation to achieve a multi effect. The fusion process is calculated in the following manner:

$$F_{Sout}=F_{smcout}+F_{seout}+F_{Gistout}$$ (19)

where ***F***_***Sout***_∈**R**^*H*×*w*×*C*^ denotes the output of the gist space features of the multi-information flow.

The first stage of the MBGSFE-Module entails employing convolutional kernels with diverse dimensions to describe ground entities that demonstrate notable variations in size. Subsequently, it employs a spatial attention mechanism to effectively capture crucial spatial features. It then introduces gist feature extraction and finally combines these three features. As a result, the inclusion of the relative positional relationship between targets is considered during the extraction of local features for scene classification, leading to an improved ability to accurately classify various scenes within a given category. The SSFE module and MBGSFE module are designed to represent the primary attributes of their respective modalities by extracting features from individual modes while maximizing dissimilarities between multiple modalities. This collaborative approach facilitates accurate classification of features.

## 3. Experiments

### 3.1 Data description

#### 1) Augsburg dataset

**Fig 5:**
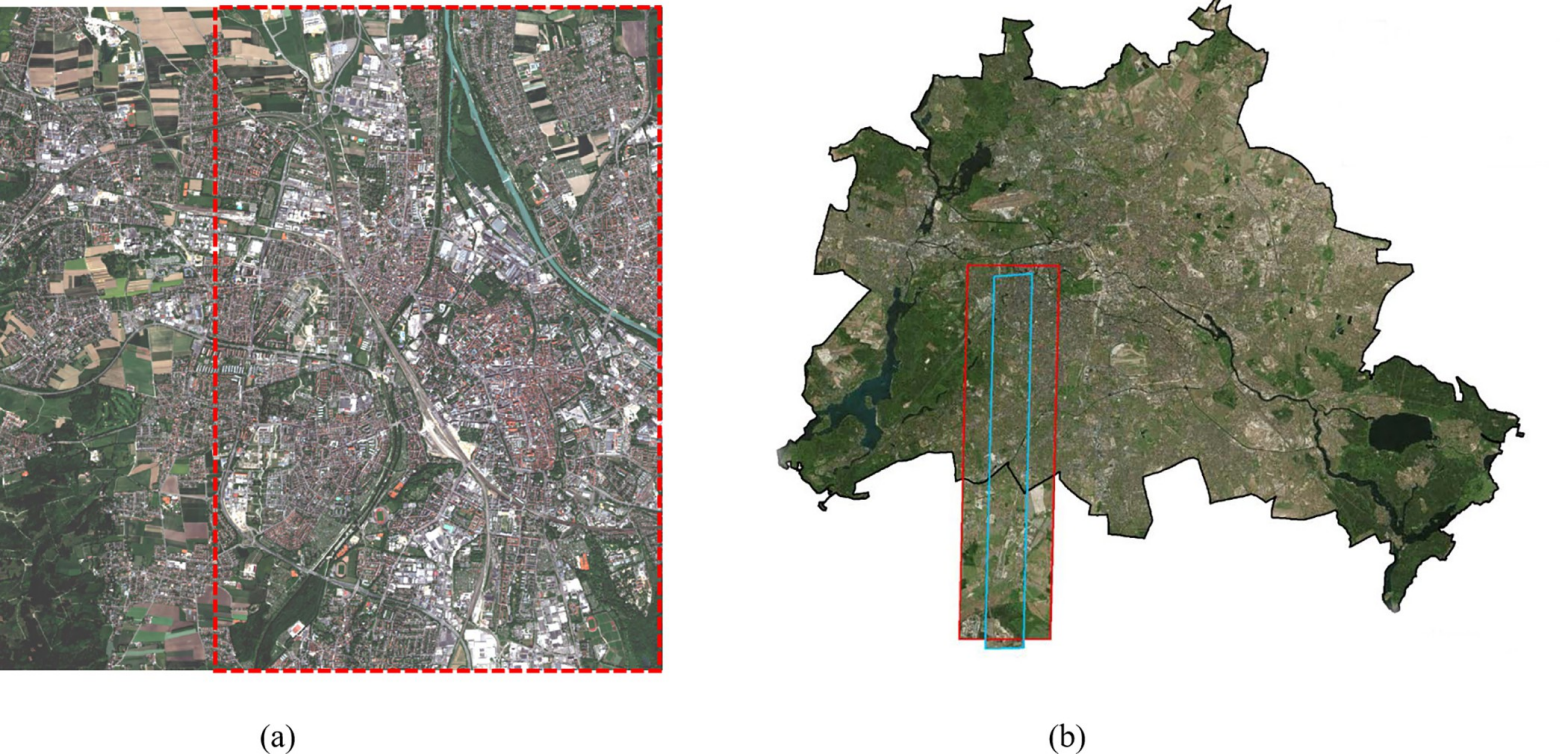
Shows the location of the study area in the experiment. (a) The Augsburg dataset study area(This image is similar but not identical to the original, Data public visit https://doi.org/10.14459/2022mp1657312 with a CC BY 4.0 license [31]). (b) The Berlin dataset study area(This image is similar but not identical to the original image, for illustrative purposes only).

This dataset comprises three distinct data sources collected in Augsburg, Germany(48°36’N, 10°93’E). The Augsburg dataset contains HSI, SAR, and Digital Surface Model (DSM) images, which are composed of HySpex sensor, Sentinel-1 satellite SAR sensor and DLR-3K system capture. In the Augsburg dataset, all images have a consistent ground sampling distance of 30m. Specifically, the Augsburg dataset has spatial dimensions of 332×485 pixels, while the HSI component comprises 180 spectral bands spanning from 0.4 to 2.5 μm in wavelength range. while the synthetic aperture SAR component comprises four features: VV intensity and VH intensity. In this paper, the data of HSI and SAR modes in Augsburg dataset are selected for fusion experiment. Table 1 provides information on the categories involved in the scenarios as well as the sizes of both training and test sets. The location of the research area in the experiment is shown in Fig 5. The Augsburg dataset can be downloaded from the website: https://mediatum.ub.tum.de/1657312.

**Table 1:** Describes the data partitioning of the Augsburg dataset.

Class No	Class Name	Training Set	Testing Set
C1	Forest	2161	11346
C2	Residential Area	4853	25476
C3	Industrial Area	456	2395
C4	Low Plants	4297	22560
C5	Allotment	92	483
C6	Commercial Area	263	1382
C7	Water	245	1285
	Total	12367	64927

#### 2) Berlin dataset

The dataset provides a comprehensive description of berlin city and its rural periphery(52°31’N, 13°2’E). It comprises an EnMAP HSI, which emulates the same area using HyMap HSI data, accompanied by corresponding Sentinel-1 SAR data. The HS image is composed of 244 spectral bands spanning from 400 to 2500 nm, covering an area of 797×220 pixels. On the other hand, dual-polarized SAR, involving four bands (VV-VH). Additionally, ground truth maps are generated based on OpenStreetMap data both training and test sets as listed in Table 2. The location of the research area in the experiment is shown in Fig 5. The Berlin dataset can be downloaded from the website: http://doi.org/10.5880/enmap.2016.002.

**Table 2:** Describes the data partitioning of the Berlin dataset.

Class No	Class Name	Training Set	Testing Set
C1	Forest	8793	46161
C2	Residential Area	42983	221232
C3	Industrial Area	3131	16435
C4	Low Plants	9485	49797
C5	Soil	2788	14623
C6	Allotment	2129	11176
C7	Commercial Area	3912	20912
C8	Water	1068	5604
	Total	74289	385940

### 3.2 Experimental setup

#### 1) Evaluation metric

This study evaluates the classification accuracy of multimodal remote sensing data by employing three widely used evaluation metrics. Its evaluation indicators are overall accuracy (*OA*), average accuracy (*AA*), and kappa coefficient (*κ*). Generally, higher values for these metrics indicate better classifier performance in RS image classification tasks. *OA*, *AA*, and *κ* provide quantitative evaluations of classification performance, with their respective calculations as follows.

$$OA=\frac{N_{c}}{N_{a}}$$ (20)

$$AA=\frac{1}{C}\sum_{i=1}^{C}\frac{N_{c}^{i}}{N_{a}^{i}}$$ (21)

and

$$\kappa =\frac{OA-P_{e}}{1-P_{e}}$$ (22)

where *N*_*c*_ denotes the number of correctly categorized samples, *N*_*a*_ denotes the number of all samples, $N_{c}^{i}$ denotes the number of correctly categorized samples in the ith category, and $N_{a}^{i}$ denotes the number of correctly categorized samples in the ith category among all samples. *P*_*e*_ in *κ* is defined as the expected prior probability and is calculated as:

$$P_{e}=\frac{N_{r}^{1}\times N_{p}^{1}+\ldots N_{r}^{i}\times N_{p}^{i}+\cdots +N_{r}^{C}\times N_{p}^{C}}{N_{a}\times N_{a}}$$ (23)

where $N_{r}^{i}$ denotes the number of true samples in each category and $N_{p}^{i}$ denotes the number of predicted samples in each category.

#### 2) Implementation details

The TensorFlow platform is utilized to implement the experimental environment for evaluating the proposed method, using a tower server, the central processing unit (CPU) utilized is the Inter Xeon Silver 4212 processor, operating at a frequency of 2.50 GHz. It is accompanied by a substantial memory capacity of 64 GB RAM and an impressive NVIDIA GeForce RTX 3080Ti graphics processing unit (GPU), equipped with a VRAM size of 16 GB. In this paper, depending on the different datasets and the number of labelled samples, about 16% of the sample data from the two datasets are randomly selected as training samples to complete the fixed hyperparameters of the network model, respectively. In all the experiments, the value of the learning rate *λ* is established as 0.001, the size of the batch block is configured as 64, while the optimizer selected is Adam, and the iteration round epoch is set to 50. In Fig 1 above, the TSFEM block, the GSFEM block, and the ResNets block in the feature extraction module are composed of a number of batch blocks, and *n* denotes the number of feature extraction batch blocks, and The entire network architecture is inspired by the layer count of ResNet18. Each ResNet Block consists of a pair of convolutional layers and in this paper, we choose Augsburg data for the hyperparameter selection comparison experiment. The experimental findings are presented in Table 3.

**Table 3:** Hyperparameter comparison results in Augsburg dataset.

*N*	9	10	11	12
OA (%)	89.56	91.62	**92.21**	92.03
AA (%)	72.65	73.35	**75.61**	75.26
*κ* (%)	87.21	89.05	**89.09**	89.45

Through the experiment, when the hyperparameter selection *n* = 11, OA highest accuracy, the overall performance of the optimal, therefore, in this article, we choose *n* = 11 as the number of batch block feature extraction phase.

The learning rate *λ* holds significant importance as a hyperparameter within the deep learning network model, influencing both the convergence of the objective function and its attainment of local optimal value. The appropriate learning rate enables the objective function to rapidly converge towards a local minimum. In the experiment, the learning rate is chosen from the candidate set {0.00001, 0.00005, 0.0001, 0.0005, 0.001, 0.005}. In this experiment, by comparing different learning rates on the Augsburg dataset, The performance of the proposed TGF-Net method is found to be satisfactory and yields the highest classification result when *λ* = 0.001, as demonstrated by the experimental outcomes presented in Table 4. Therefore, *λ* = 0.001 is chosen as the experimental learning rate in this paper.

**Table 4:** Learning rate comparison on the Augsburg dataset.

*λ*	0.00001	0.00005	0.0001	0.00005	0.001	0.005
OA (%)	87.26	88.43	89.51	91.43	**92.21**	92.04
AA (%)	70.45	72.15	73.73	75.06	**75.61**	75.11
*κ* (%)	85.21	86.12	87.84	88.25	**89.09**	89.01

#### 3) Ablation experiments

In this study, we employ the Augsburg dataset as a case study to assess each module in analyzing the overall network structure. The detailed outcomes are presented in Table 5. It is evident from the table that the optimal outcomes are achieved, TSFEM, GSFEM, and ResNet module. The module for feature reorganization has the capability to intelligently improve the features by breaking them down using non-negative matrices and self-attention mechanism, followed by resolving similarity calculations. It decreases the repetition of redundant information from different modes in a particular setting, it amplifies the excellence of characteristics. TSFEM effectively chooses the input for the transformer and determines the sequence of channel features in hyperspectral images, enabling it to accurately capture the spectral characteristics present in such images. The GSFEM is created to tackle the issue of significant variations in large remote sensing targets by utilizing various sizes and incorporating gist features. This enables the representation of multiple scales within a single space, facilitating the network’s comprehension of feature interrelationships. It has been experimentally confirmed that the enhancement in overall classification accuracy is solely attributed to the collaboration among exclusively these four modules.

**Table 5:** Classification results of Augsburg data for different module combinations.

FR	TSFEM	GSFEM	RESNET	OA (%)	AA (%)	Kappa (%)
			✓	83.12	70.45	83.26
✓			✓	85.36	71.12	86.02
	✓		✓	89.12	71.23	86.15
		✓	✓	89.45	72.38	86.28
✓	✓		✓	90.14	72.85	87.54
	✓	✓	✓	90.75	72.96	88.02
✓	✓	✓		91.03	73.35	88.71
✓	✓	✓	✓	**92.21**	**75.61**	**89.09**

### 3.3 Experimental results and analysis

To authenticate the classification effect of the TGF-Net network proposed in this paper, multiple advanced algorithms for classifying multi-channel remote sensing data have been chosen to conduct both quantitative and qualitative comparisons, such as Two-branch CNN [32], S2FL [33], depth wise feature interaction network (DFINet) [34], coupled CNN (CPCNN) [35], ACLCNN [36], CMR-Net [26] and TGF-Net. Among them, TGF-HSI represents the classification accuracy of the TGF-Net method proposed in this paper under a single HSI mode.

#### 1) Quantitative comparison on the Augsburg dataset

Table 6 presents the quantitative outcomes of various approaches in terms of evaluating the metrics OA, AA, and κ. Overall, the classification performance of various classification techniques on features belonging to forest, residential, and low plants categories is considerably high, with OA values consistently exceeding 90%. The TGF-Net classification method of this paper has improved its OA by more than 5% compared to the Two-branch CNN classification method. It suggests that the TGF-Net approach employed in this study possesses the capability to derive more distinctive feature representations and effectively capture the inherent correlation among diverse modalities. Specifically, the OA values of TGF-Net are improved by +5.38%, +8.35%, +3.33%, +4.00%, +1.01% and +0.67%, respectively, compared with other deep learning classification methods. In addition, compared with the classification results of other methods, TGF-Net in this paper achieved significant classification performance on C1, C2, C4, C5 and C6, with improved classification accuracy. The overall classification accuracy of the single-modal HSI data classified by the TGF-Net method is lower than that of the multi-modal TGF-Net method, and the accuracy of the multi-modal fusion TGF-Net method is about 4.00% higher than that of the single-modal. Thus, The results suggest that TGF-Net exhibits superior performance compared to alternative approaches and attains the most optimal classification outcomes in the fusion and classification of multimodal data.

**Table 6:** Quantitative comparison of different methods in terms of OA, AA, and κ on the Augsburg datasets.

Category	Two-branch CNN	S2FL	DFI-Net	CPCNN	ACLCNN	CMR-Net	TGF-HSI	TGF-Net
C1	93.76±01.04	88.80±02.25	97.10±01.21	93.42±00.35	97.23±00.34	98.15±00.76	97.21±00.28	**98.35±00.23**
C2	94.19±01.63	89.36±02.28	95.56±02.04	96.28±01.28	98.34±01.02	96.42±00.51	91.76±00.52	**98.54±00.31**
C3	58.73±01.75	45.90±02.26	55.26±01.16	52.57±02.05	**72.35±01.01**	71.40±00.28	72.30±00.42	72.10±01.32
C4	85.51±02.28	87.53±03.02	88.35±01.82	89.62±01.63	89.59±00.46	90.17±01.46	90.02±00.25	**90.32±00.34**
C5	51.86±01.06	68.64±02.16	60.38±01.27	63.15±01.62	67.86±01.23	64.60±01.23	40.46±00.58	**69.58±00.28**
C6	28.35±02.08	8.97±02.44	30.21±01.64	10.24±02.04	26.62±01.84	39.80±00.80	30.25±00.57	**40.15±01.37**
C7	49.37±01.64	47.65±01.72	51.12±01.26	40.68±01.42	50.12±01.09	**63.89±01.29**	60.25±00.79	60.26±00.87
OA (%)	86.83±01.24	83.86±02.06	88.88±01.52	88.21±01.41	91.20±01.10	91.54±00.87	88.97±00.38	**92.21±00.52**
AA (%)	65.97±01.10	62.40±01.62	68.28±01.44	63.71±01.31	71.73±00.84	74.92±01.16	68.89±00.29	**75.61±01.04**
κ (%)	81.91±01.29	78.03±01.72	84.59±01.31	83.64±01.73	87.70±01.28	88.11±00.75	83.09±00.31	**89.09±00.87**

#### 2) Visual comparison on the Augsburg dataset

**Fig 6:**
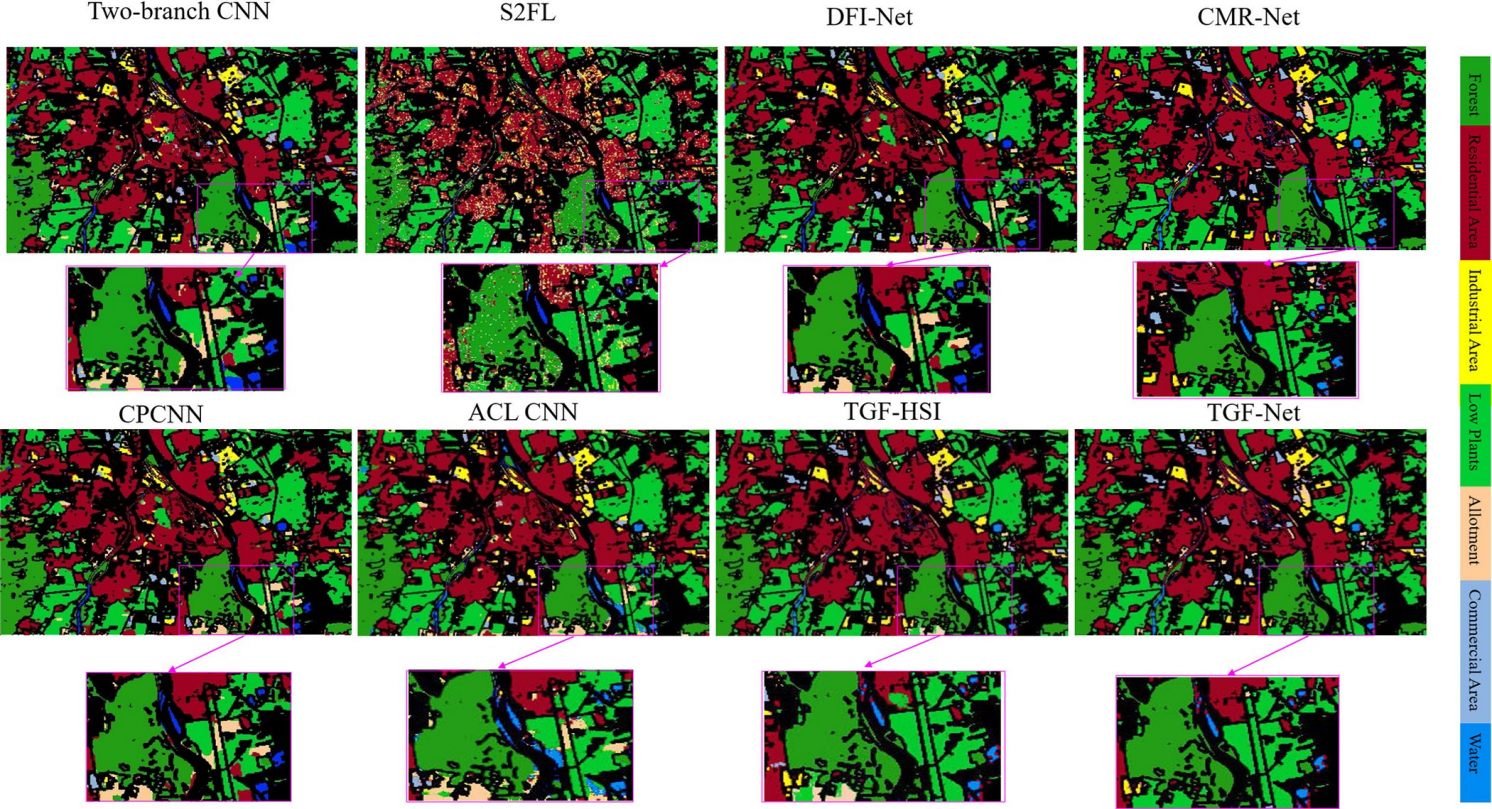
Classification maps of different multi-modality methods on the Augsburg dataset (Tow—branch CNN (86.83%), S2FL (83.86%), DFI-Net (88.88%), CPCNN (88.21%), ACLCNN (91.20%), CMR-Net (91.54%), TGF—Net (92.21%)).

Fig 6 shows the comparison effect graph between the TGF-Net method proposed in this paper and other multimodal remote sensing data classification methods. Through visual observation, it has been observed that the land cover labels produced by TGF-Net’s classification maps exhibit enhanced realism and improved spatial aggregation outcomes and the generated classification maps are smoother and less noisy. The deep learning approach performs well on the classification maps, especially the boundary delineation between vegetation, such as forests and low-growing plants. In order to illustrate the classification effect more intuitively and clearly, some areas in the classification map are enlarged. The classification effect of the proposed TGF-Net method for single modal HSI data is inferior to that of the multi-modal TGF-Net method, especially in the classification effect of C5 (Allotment) is worse than that of other classification methods. The TGF-Net classification method is able to search for effective spatial and spectral information from highly complex scenes, which produces feature classification results that are closer to the real ones. Its classification visualization results on the Augsburg dataset are shown in Fig 6.

#### 3) Quantitative comparison on the Berlin dataset

Table 7 quantitatively presents the evaluation indexes for OA, AA, and κ were utilized to assess the classification outcomes of various methods. In general, the TGF-Net classification method outperforms various other classification methods in terms of accuracy and performance. Its OA value is increased by +8.30%, +18.30%, +8.12%, +9.64%, +2.56% and +2.24%, respectively. It can be fully demonstrated that the TGF-Net method can extract more discriminative feature representations and achieve high-quality feature fusion, which better captures the intrinsic relationship between different modalities. At the same time, it is evident that the convolutional neural network-based method outperforms the S2FL classification method in terms of classification performance. The classification accuracy is improved by at least 8%. Moreover, the classification accuracy of TGF-Net is higher than other classification methods on C2, C3, C4, C7 and C8. There exists a presence of disturbance during the process of feature extraction and fusion, resulting in the inability to acquire consistent features. The classification accuracy is inferior to CPCNN method and ACLCNN method. The overall classification accuracy of the single-modal HSI data classified by the TGF-Net method is lower than that of the multi-modal TGF-Net method, and the accuracy of the multi-modal fusion TGF-Net method is about 3.50% higher than that of the single-modal. Hence, this suggests that TGF-Net surpasses alternative approaches and attains superior outcomes in the fusion and classification of multimodal data.

**Table 7:** Quantitative comparison of different methods in terms of OA, AA, and κ on the berlin datasets.

Category	Two-branch CNN	S2FL	DFI-Net	CPCNN	ACLCNN	CMR-Net	TGF-HSI	TGF-Net
C1	81.14±02.18	79.52±03.54	80.29±02.06	78.35±02.13	78.29±01.67	**89.25±01.22**	80.41±00.56	82.27±01.38
C2	61.15±02.64	49.41±02.35	61.93±01.89	60.29±02.36	69.71±01.46	68.56±00.88	68.14±00.62	**72.34**±**00.96**
C3	57.68±03.08	45.18±03.26	47.44±02.61	61.21±03.32	65.26±02.10	68.56±01.43	60.10±01.13	**75.80**±**00.53**
C4	84.03±01.84	70.50±02.32	80.01±01.67	75.67±02.02	86.74±01.18	84.79±00.65	87.12±00.85	**87.32**±**01.65**
C5	79.80±01.18	81.47±01.46	77.54±02.05	**85.69**±**02.10**	82.37±00.73	85.45±00.91	82.02±01.23	82.08±01.54
C6	70.81±02.05	61.31±02.23	73.42±01.64	**75.62**±**01.03**	75.01±02.06	65.41±01.12	73.21±01.28	75.36±00.87
C7	34.17±03.06	29.63±01.78	49.11±02.03	37.16±01.66	37.74±01.85	38.52±00.52	38.11±00.76	**38.56**±**01.36**
C8	78.53±01.86	57.24±02.34	77.59±01.17	71.52±01.26	81.71±01.47	73.63±00.49	82.56±00.45	**82.72**±**00.62**
OA (%)	66.06±02.09	56.06±01.48	66.24±02.03	64.72±01.76	71.80±01.64	72.12±01.06	70.94±00.38	**74.36**±**00.53**
AA (%)	68.41±01.87	59.28±01.23	68.42±01.86	68.19±01.41	72.10±01.32	71.77±00.83	71.46±00.57	**74.56**±**00.49**
κ (%)	53.93±02.06	42.46±01.53	53.98±02.12	52.42±02.03	60.34±02.08	62.51±01.24	60.77±00.46	**64.69**±**00.76**

#### 4) Visual comparison on the Berlin dataset

**Fig 7:**
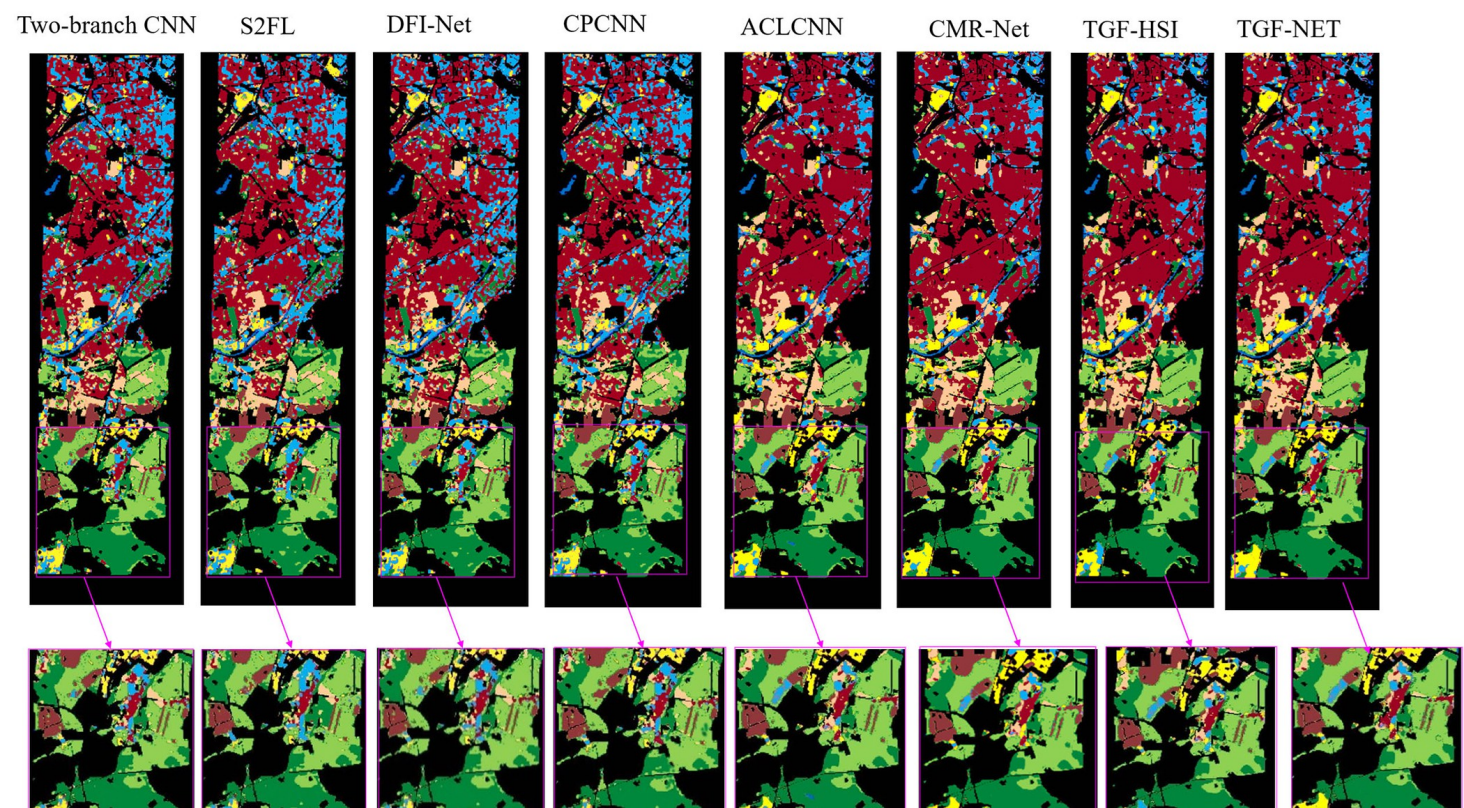
Classification maps of different multi-modality methods on the Berlin dataset (Tow—branch CNN (66.06%), S2FL (56.06%), DFI-Net (66.24%), CPCNN (64.72%), ACLCNN (71.80%), CMR-Net (72.12%), TGF—Net (74.36%)).

The comparison plot in Fig 7 illustrates the classification performance of the proposed TGF-Net method in contrast to other classification methods. After careful observation, it is evident that the classification map generated by TGF-Net exhibits superior performance, especially the classification effect maps of C1(forest), C2(residential area) and C3(industrial area) are better than those of other methods, which can produce a clearer classification effect. Among them, there are many noises in several classification maps of CPCNN, DFI-Net, Two-branch CNN,S2FL and CMR-Net, and some C2(residential area) are misclassified as C7(commercial area). In order to illustrate the classification effect more intuitively and clearly, some areas in the classification map are enlarged. Through the proposed TGF-Net method, the classification effect of single modal HSI data is inferior to that of the multi-modal TGF-Net method, and the multi-modal method is effective for C1(Forest) and C4(Low Plants) is better than that of a single modality. The proposed TGF-Net classification method can generate clearer classification effect maps and produce land cover distribution closer to the real scene. Its classification visualization results on the berlin dataset are shown in Fig 7.

#### 5) Comparison of different proportions of training samples on different methods

**Fig 8:**
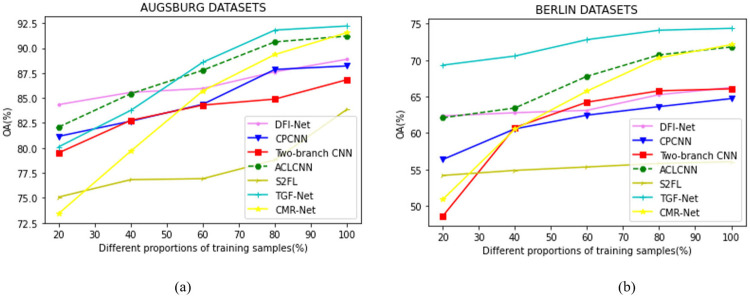
OA values of different classification methods with different proportions of training samples. (a) Augsburg dataset. (b) Berlin dataset.

In order to verify the stability and robustness of the proposed method in the case of small samples, different proportions of training sample data are used to conduct experiments to prove the performance of the proposed method. We conduct comparative experiments and analyze the experimental results. It can be seen from the experimental results in Fig 8 that the classification methods based on deep learning are relatively less sensitive to the number of samples, and the ACLCNN and the proposed TGF-Net methods have smoother accuracy trends under different proportions of training samples, and their OA is also higher than other classification methods. The proposed method only uses less than 20% of the training sample data to maintain good performance. Therefore, increasing the proportion of training data has little impact on the accuracy improvement, which indicates that the transformer and Gist convolution-based method can reveal more valuable features.

#### 6) Computational complexity comparison

In this paper, the time complexity of Two-branch CNN, S2FL, DFINet, CPCNN, ACLCNN,CMR-Net and the comparison and analysis of the approach presented in this paper are demonstrated in Table 8. Since the TGF-Net network proposed in this paper designs different feature extraction networks for different modal data in the stage of merging features, the computational complexity of the proposed method is comparatively higher due to limited time efficiency during feature fusion, in contrast to other methods.

**Table 8:** The time complexity of the experimental test dataset.

Classification Method	Time(s) on berlin	Time(s) on Augsburg	Complexity Calculation
Two-branch CNN	600.81	308.51	O(C^2^)
S2FL	520.26	280.28	O(C^2^+C)
DFINet	481.18	279.68	O(3C^2^+5C)
CPCNN	500.15	272.32	O(3C^2^+C)
ACLCNN	468.42	258.45	O(8C^2^+3C)
CMR-Net	459.65	263.48	O(4C^2^+4C)
TGF-Net	480.85	300.64	O(2C^2^+11C)

## 4. Discussion and analysis

The TGF-Net classification method proposed in this paper is still based on CNN. Compared with other classification methods, the multi-modal data of HSI and SAR are firstly decomposed by non-negative matrix decomposition before feature extraction, which can be used to quickly capture and identify image features and is more in line with human visual perception. At the same time, the similarity calculation and self-attention feature enhancement are performed on the data after feature decomposition, so that the original feature data can be reconstructed into common feature and unique feature matrices. However, some other classification methods only divide the original features into blocks and input the blocked features into the feature extraction module without reconstructing the features. Secondly, in the feature extraction stage, such as Two-branch CNN method, S2FL method and CPCNN method, these methods only use the CNN-based method to extract HSI and SAR features respectively, which has more calculation parameters and will have overlapping feature information. In the feature extraction stage, DFINet method and ACLCNN method extract effective spectral and spatial features by encoding and decoding HSI and SAR features, cross-attention calculation and other ways. In the feature extraction stage, the network design is complex and has information redundancy. However, in the feature extraction stage, the TGF-Net method proposed in this paper sets up a feature extraction module for HSI, a feature extraction module for SAR and a common feature extraction module, which makes the network structure design simple and reduces the redundancy of features. In the feature fusion stage, except the ACLCNN method, which decodes the classified features and then outputs the classification results, the other methods output the classification results after full connection. In short, through the comprehensive analysis of several classification methods, the proposed method has been improved and improved in network structure design and performance compared with other methods.

## 5. Conclusion

In order to better and more efficiently classify multimodal remote sensing data, This research presents TGF-Net, an innovative network that integrates transformer and Gist CNN feature fusion methods for the classification of multimodal HSI and SAR images. The feature extraction of HSI and SAR images is enhanced in the network architecture by incorporating a transformer-based Spectral Feature Extraction Module and a Gist-based spatial feature extraction module. FR module Combining self-attention mechanisms and positive matrix factorisation, effectively eliminates information redundancy among multimodal images while simultaneously extracting their unique and shared features. The TSFEM captures the sequencing of multiple channels, while the GSFEM determines the relative spatial relationships. As a result, TGF-Net effectively represent multimodal data features and their classification accuracy is validated through experiments. Despite its advantages in classifying multimodal remote sensing data, further development is to enhance its practicality and applicability. However, the network structure of TGF-Net proposed in this paper is slightly cumbersome and complex, because the network designs different feature extraction networks by feature extraction of different modes, so there is a situation that the network is not stable enough. In the later research work, we will simplify and optimize the network structure, not only to improve the efficiency, but also to maintain the accuracy. In the near future, we plan to optimize and prune the network to enhance efficiency without compromising accuracy. In our future research, we will focus on developing a fusion network that is more efficient to overcome the performance bottleneck of the TGF-Net.
